# Validation of biomarkers and clinical scores for the detection of uterine leiomyosarcoma: a case-control study with an update of pLMS

**DOI:** 10.1186/s12885-024-13396-y

**Published:** 2025-01-08

**Authors:** Marcus Vollmer, Günter Köhler, Julia Caroline Radosa, Marek Zygmunt, Julia Zimmermann, Martina Köller, Christine Seitz, Helena Bralo, Marc Philipp Radosa, Askin Cangül Kaya, Johann Krichbaum, Erich-Franz Solomayer, Lars Kaderali, Zaher Alwafai

**Affiliations:** 1 Institute of Bioinformatics, University Medicine Greifswald, Felix-Hausdorff-Str. 8, Greifswald, 17475 Germany; 2 Department of Obstetrics and Gynecology, University Medicine Greifswald, Sauerbruchstr., Greifswald, 17475 Germany; 3 Department of Gynecology, Obstetrics and Reproductive Medicine, Saarland University Hospital, Kirrbergerstr., Homburg/Saar, 66421 Germany; 4 Department of Gynecologic Surgery, Hospital Sachsenhausen, Schulstr. 31, Frankfurt am Main, 60594 Germany; 5 Department of Gynecology and Obstetrics, University Hospital of Munich (LMU), Marchioninistr. 15, Munich, 81377 Germany; 6 Department of Gynecology and Obstetrics, Hospital Bremen Nord, Hammersbecker Str. 228, Bremen, 28755 Germany; 7 Outpatient Department, Gynmünster, VAAO, Hohenzollernring 57, Münster, 48145 Germany

**Keywords:** Uterine leiomyosarcoma, Leiomyoma, Diagnostics, Clinical score, Lactate dehydrogenase, Neutrophil-to-lymphocyte-ratio, Platelet-to-lymphocyte-ratio

## Abstract

**Background:**

The diagnosis of rare uterine leiomyosarcoma (uLMS) remains a challenge given the high incidence rates of benign uterine tumors such as leiomyoma (LM). In the last decade, several clinical scores and blood serum markers have been proposed. The aim of this study is to validate and update the pLMS clinical scoring system, evaluating the accuracy of the scoring system by Zhang et al. and examining the discriminatory ability of blood markers such as serum lactate dehydrogenase (LDH), neutrophil-to-lymphocyte ratio (NLR), and platelet-to-lymphocyte ratio (PLR).

**Methods:**

In a case-control study, 90 new uLMS from the DKSM consultation registry and 659 prospectively recruited LM cases from the Saarland University Hospital were used for validation. Welch’s t-test and Hedges’ *g* were used to evaluate blood markers and optimal thresholds and diagnostic odds ratios were calculated. Scoring systems were compared using receiver operating characteristics and proposed diagnostic cut-offs were reviewed. Missing values were imputed by random forest imputation to create the updated scoring system ‘pLMS2’ using penalized logistic regression based on the pooled data sets of 384 uLMS and 1485 LM.

**Results:**

pLMS achieved an AUC of 0.97 on the validation data, but sensitivity and specificity varied at the proposed thresholds due to a shift in the score distributions. 43 uLMS and 578 LM were included in the comparison of pLMS with Zhang’s scoring system, with pLMS being superior (AUC 0.960 vs 0.845). LDH, NLR, and PLR achieved a diagnostic odds ratios of 18.03, 8.64 and 4.81, respectively. pLMS2 is based on subscores for menopausal status interacting with age, tumor diameter, intermenstrual bleeding, hypermenorrhea, dysmenorrhea, postmenstrual bleeding, rapid tumor growth, and suspicious sonography.

**Conclusions:**

Validation of the pLMS shows stable discriminatory ability as expressed by AUC, although caution should be taken with cut-off values, as sensitivity and specificity may vary. Data collection of the updated clinical score pLMS2 remains simple and convenient, with no additional cost. The proposed thresholds of 1.5 and 5.5 can be used as a guide to avoid unnecessary or inappropriate surgery and to make the use of further diagnostic measures cost-effective. LDH, NLR and PLR provide further evidence to differentiate uLMS from LM in conjunction with clinical data.

## Introduction

### Relevance

The preoperative diagnosis of highly malignant uterine leiomyosarcoma (uLMS) remains a major clinical challenge. The main problem is the discrimination from benign uterine masses. Uterine masses are typically benign leiomyomas (LM) that arise most commonly between menarche and menopause with estimated cumulative incidence rates exceeding 80% in black women and 70% in white women by age 50 [1]. Treatment options for LM include total or supracervical hysterectomy or myomectomy, performed with or without morcellation [2]. In contrast, for uLMS, the appropriate surgery is total hysterectomy without injuring the uterus [3]. Hence uLMS are often coincidental findings at the time of hysterectomy and the diagnosis is mostly made postoperative [4, 5]. As a result, uLMS are often operated on like LM, which is often accompanied by morcellation, which may lead to shorter survival time in the case of uLMS [6]. An older systematic literature review and meta-analysis of four studies with 202 patients [7] and another study from 2017 [6] with 125 patients found an increased locoregional recurrence rate with worse overall survival after a morcellation has been performed. However, the distant metastasis-free interval was not changed by morcellement. Nevertheless, more recent studies with significantly higher case numbers (n=301 [8] and n=152 [9]) have shown that morcellation only worsens locoregional recurrence-free interval, but not overall survival. The distant metastasis-free interval was even longer, which cannot be explained at this time. However, locoregional recurrence with possible subsequent surgery, chemotherapy or radiotherapy must be avoided. This can only be achieved by preoperative differentiation between LM and uLMS.

At the stage of the review by George et al. in 2018 the authors concluded that “Unfortunately, in uLMS, no single imaging criterion can reliably distinguish a benign uterine tumor from one that is malignant” [10], which makes the incidental findings comprehensible.

### Preoperative classification

Several studies have been published dealing with preoperative tumor classification (uLMS diagnosis). Some important findings are summarized here.

Akazawa et al. published 2021 a systematic review on the application of artificial intelligence in gynecologic cancers [11]. Only three studies on uterine sarcoma were identified, of which two used imaging data and one used value-based data. The image-based methods were trained on magnetic resonance imaging (MRI) with useful AUCs (area under the receiver operating characteristic) of 0.972 [12] and AUC of 0.92 [13]. The problem with these studies is the small number of uterine sarcomas and LM controls (9 in Malek, 3 uLMS in Nakagawa out of a total of 11 uterine sarcomas). Multivariable logistic regression with feature selection resulted in a final model with mean apparent diffusion coefficient and normalized T2-weighted imaging (T2W) as predictor variables used by Nakagawa. The regression model may be overfitted, as the recommended number of events per variable usually only allows the inclusion of a single predictor. Proof of generalizability with external validation sets is also pending. Due to the high incidence of LM on the one hand and the very low incidence of uLMS on the other hand, MRI is not suitable for the primary differentiation of LM and LMS for economic reasons alone. Therefore, a risk assessment based on symptoms or clinical findings must be performed prior to its use. The third is a value-based model (published by us) that was trained with XGboost and Random Forest and compared with a multivariable logistic regression [14]. We did not find superior results to the classical statistical method of logistic regression, which has the advantage that hypothesis testing can be performed and the effect sizes are expressed as odds ratios with confidence intervals. The preoperative clinical score, which we called pLMS, was trained on 80% of an outstanding number of 293 uLMS and achieved a mean cross-validated AUC of 0.969 in the training set and an AUC of 0.968 in the test set. Key features were bleeding symptoms: intermenstrual bleeding, hypermenorrhea, dysmenorrhea, postmenstrual bleeding, suspicious sonography and the maximum tumor diameter. An initial assessment of the proposed cut-off points was performed with LMS patients from the NOGGO-REGSA registry [15]. The findings indicated that the “indicator for leiomyosarcoma” (pLMS${>}1$) was accurate in only 39.1% of patients, thereby raising questions about the reliability of the score. Consequently, the authors did not recommend its use in clinical practice. As the cases of LM were not included in the study, the authors were unable to draw any conclusions regarding the diagnostic ability of the pLMS. In addition, the methodology employed to address missing data was deemed insufficient by our team, see conversation [16, 17].

Preoperative serum markers may also be useful for disease classification. In a retrospective study, Zhang et al. discussed the diagnostic value of cancer antigen 125 (CA125), lactate dehydrogenase (LDH), and human epididymal protein 4 (HE4) for the preoperative diagnosis of LMS [18]. The most promising marker was LDH, which yielded an AUC of 0.81 compared with 102 patients with degenerated uterine leiomyoma. Combining HE4 or CA125 increased the AUC by 6%. Suh et al. examined blood markers and suggests that postmenopause, white blood cell (WBC) count, absolute neutrophil count, C-reactive protein, and neutrophil-to-lymphocyte ratio (NLR) serve as cost-effective, and widely available biomarkers [19]. Increased odds of uLMS (odds ratios between 2 and 10) reported 2020 by a Liverpool group were postmenopausal status, symptoms of pressure, postmenopausal bleeding, neutrophil count $\ge 7.5 \times 10^9/L$, hemoglobin level <118 g/L, endometrial biopsy results of cellular atypia or neoplasia, and tumor size $\ge 10$ cm [20]. In the same year Zhang et al. created a preoperative score based on age, tumor size, NLR, platelet count, and LDH [21]. From a matched control study including 117 women who underwent hysterectomy, a risk prediction model had an AUC of 0.83 [22]. Other value-based studies have had too few participants for their findings to be generalizable: Wojdat & Malanowska presented a 10-variable myomscore with intuitive cut-offs based on 6 LMS cases only [23]. The revised PREoperative Sarcoma Score (rPRESS), is based on 63 patients with suspected uterine sarcoma, of which only 15 had uterine sarcoma (9 uLMS) [24]. rPRESS includes LDH and has a positive predictive value of 63% and a negative predictive value of 93%.

While all of these AUC values appear promising, the strong imbalance of uLMS vs. LM, would lead to a considerable number of false positives, given the low prevalence rates (see Halaska et al. [25]). We are therefore focusing our efforts on identifying phenotypic differences between uLMS and LM. This will provide the evidence needed to justify the use of advanced blood analysis, imaging, and potential genetic options in uLMS diagnosis.

## Methods

### Aims

The main objective of this study is to validate and the update the clinical score system pLMS [14] on newly unseen data of uLMS and LM cases. Secondary objectives are to test the transferability of the score system of Zhang et al. [21], originally trained on Chinese patients, to our predominantly German population and to explore the discriminatory ability of blood markers such as LDH, NLR, and platelet-to-lymphocyte ratio (PLR).

### Patients and study design

This is a case-control study that uses unseen new data for the validation part. Cases include 90 predominantly retrospective uLMS patients treated between 1st January 2019 and 31st December 2020 taken from a registry – the counseling database of the German Clinical Center of Excellence for Genital Sarcomas and Mixed Tumors (DKSM, University Medicine Greifswald, Germany). Controls are 659 histologically proven and prospectively collected LM patients receiving surgery between 1st January 2015 and 31st December 2020 at the Saarland University Hospital, Germany.

To update the pLMS scoring system and to validate and explore further diagnostic tools, we pooled the new data with the original data from the previous multicenter cohort study (Köhler et al. [14]). In that study “826 patients with LM were recruited, of them 577 were collected prospectively from a clinical institution (Center of Minimally Invasive Surgery at the hospital Sachsenhausen, Germany) and 249 (192 retrospectively) from an outpatient center (Velener Working Group on Ambulatory Surgery at Gynmünster, Münster, Germany)” from 2014 to 2018. 293 histologically proven uLMS has been used from the counseling database of the DKSM from 1st August 2010 to 31st December 2018. 1 additional uLMS case was added to the consultation database with a delay, which led to 294 documented uLMS before 2019.

Data recorded for each patient included clinical/laboratory variables and sonographic findings: Age at the time of surgery, maximum tumor diameter, menopausal status, suspicious sonography (as defined in our previous work [14]), rapid growth of the tumor (condition described from the medical records by the attending gynecologist), solitary tumor, symptoms (low abdominal pain, feeling of abdominal pressure or pollakiuria), anamnestic tamoxifen exposure, prior surgery due to mitotically active leiomyoma, leiomyoma with bizarre nuclei and smooth muscle tumors of uncertain malignant potential, and failure of medical therapy (ulipristal acetate) of LM. Intermenstrual bleeding, hypermenorrhea and dysmenorrhea were recorded in premenopausal women and postmenopausal bleeding in postmenopausal women. In addition, the preoperative LDH values, NLR, and PLR were obtained from the medical records when available.

**Fig. 1:**
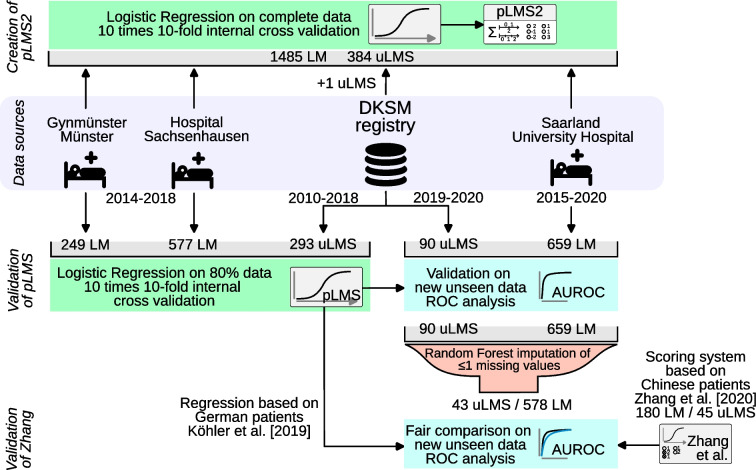
Flowchart of patient population and data flow for training and validation

Patients with a known malignant tumor were excluded. Laboratory measurements were not taken into account in patients with an acute or chronic inflammatory disease. Written informed consent was obtained from all participants and institutional review boards approved this study. This study is reported in accordance with the TRIPOD guidance for transparent reporting of prediction models [26, 27]. A flowchart of this case-control study is given in Fig. 1.

### Statistical analysis

Patient characteristics are presented in aggregated form, stratified by tumor type and data source. Continuous variables are expressed as mean and standard deviation (SD), categorical variables as absolute numbers and percentages for each level. The total proportion of missing data is expressed as a percentage. Tumor diameter and serum markers were log-normally distributed and compared by Welch’s t-test on log-scale. Hedges’ *g* was calculated as a measure of effect size and the distribution is visualized in grouped boxplots.

Optimal cut-offs were calculated for tumor size, age in interaction with menopause, and serum markers (on log2-scale) by maximizing the sum of sensitivity and specificity in 1000 bootstrap samples [28]. The average AUC (area under the receiver operating characteristic [ROC]), sensitivity and specificity and their standard deviations were calculated from all out-of-bag bootstrap samples. Diagnostic odds ratios were calculated from the average out-of-bag sensitivity and specificity. In addition, mean and standard deviation of the optimal cut-offs were determined. Boxplots were generated for continuous predictors for uLMS and the number of available values was reported.

The pLMS classification performance was assessed by applying the regression formula of pLMS [14] to patient data to compute the predictions of the unseen validation set. The ROC curve and the AUC were assessed and compared to the ROC curve of original training set. DeLong confidence intervals for the AUC were calculated [29].

To evaluate the scoring system of Zhang et al. in comparison to pLMS, we used 340 complete cases (323 LM, 17 LMS) with no missing values in the predictor variables (age, tumor diameter, NLR, LDH and platelets). Missing data patterns were screened and an additional 281 incomplete cases (255 LM, 26 LMS) with only one missing value were incorporated into the dataset. Two imputation methods were subsequently employed to fill the remaining missing values: random forest imputation (RF) and predictive mean matching (PMM) [30]. Here we also used clinical and paraclinical variables utilized for pLMS calculations. In the same patients, pLMS was also calculated and paired results were presented as Boxplots. As before, DeLong confidence intervals were calculated for the AUC in order to facilitate a comparison of the classification performance.

For the updated scoring system ‘pLMS2’ a multivariable penalized logistic regression was trained using all available variables in 10 independent runs of tenfold cross-validation to obtain robust estimates of the regression coefficients. The regularization parameter for the *L*2 norm of the coefficients was set to $\lambda {=}0.5$. Missing values were previously imputed by random forest imputation [30]. The $\beta$-coefficients of the final regression formula (estimated logits) were used to find a user-friendly integer-based score system. Here we searched for a small factor *c* where $c\beta$ is close to integers. For finding an optimal *c*, we evaluated the root mean squared error between the exact estimates $c\beta$ and the rounded values $\lfloor c\beta \rceil$^1^.

Corrected relative risks ($RR_c$) with 95% confidence intervals (CI) were computed for the exact coefficients to assess the multivariable adjusted classification performance of each predictor.

Two diagnostic cut-offs with either good sensitivity or specificity were proposed. The risk of uLMS was estimated empirically using the cumulative distributions of pLMS2 scores and presented in line charts for different hypothetical incidence rates of uLMS in LM surgery.

Statistical analysis was performed in R v4.2.0 [31]. Additional statistical packages used were: mice 3.13.0 [30], caret 6.0–94 [32], stepPlr 0.93 [33], cutpointr 1.1.2 [28], pROC 1.17.0.1 [34].

**Table 1:** Preoperative clinical characteristics of cases (uLMS) and controls (LM) of training and validation data mit proportion of missing data

	Training data (2010–2018)		Validation data (2019–2020)		Missing
	LM (N=826)	uLMS (N=294)	LM (N=659)	uLMS (N=90)	(%)
Retrospective cases (%)	192 (23.2)	266 (90.5)	0 (0.0)	84 (93.3)	
*Continuous variables, mean (SD)*					
Age at surgery [years]	43.29 (7.20)	54.12 (10.91)	42.76 (9.18)	52.70 (11.80)	–
Tumor diameter [cm]	5.49 (2.55)	10.46 (5.71)	5.40 (2.67)	11.42 (5.61)	–
LDH [*U*/*L*]	198.36 (35.91)	394.66 (314.75)	239.00 (83.69)	361.71 (205.22)	63.5
NLR	2.30 (1.14)	4.34 (2.31)	–	5.72 (5.55)	69.0
PLR	164.68 (68.98)	226.39 (77.65)	–	300.77 (201.29)	74.8
Platelets [$\times 10^9/L$]	279.39 (75.26)	324.85 (99.39)	283.16 (67.83)	368.96 (170.68)	50.1
*Continuous variables, median (lower – upper quartile)*					
Tumor diameter [cm]	5.0 (3.7–7.0)	9.0 (7.0–12.9)	5.0 (3.0–7.0)	10.0 (8.0–13.0)	–
LDH [*U*/*L*]	193 (175–217)	266 (204–484)	211 (192–235)	330 (206–420)	63.5
NLR	2.09 (1.59–2.79)	3.42 (2.98–4.83)	–	3.9 (2.7–5.6)	69.0
PLR	152 (120–194)	213 (175–285)	–	219 (191–351)	74.8
Platelets [$\times 10^9/L$]	269 (232–318)	299 (271–378)	277 (234–322)	314 (284–427)	50.1
*Categorical variables, n (%)*					
Postmenopause	32 (3.9)	170 (57.8)	55 (8.3)	40 (44.4)	–
Intermenstrual bleeding^a^	86 (10.4)	58 (19.7)	66 (10.0)	27 (30.0)	0.1
Hypermenorrhea^a^	480 (62.4)	33 (11.2)	299 (45.5)	15 (16.7)	3.2
Dysmenorrhea^a^	278 (36.2)	6 (2.0)	271 (41.2)	0 (0.0)	3.1
Postmenopausal bleeding^b^	6 (0.7)	82 (27.9)	18 (2.7)	20 (22.2)	–
Tamoxifen exposure	0 (0.0)	4 (1.4)	1 (0.2)	3 (3.3)	2.8
Symptoms^c^	409 (49.5)	132 (44.9)	28 (4.2)	39 (43.3)	–
Failed medical or interventional therapy of myoma	26 (3.1)	11 (3.7)	16 (2.4)	1 (1.1)	–
Prior surgery of atypical smooth muscle tumor(%)	7 (0.8)	7 (2.4)	0 (0.0)	2 (2.2)	–
Solitary tumor	335 (40.6)	161 (54.8)	327 (49.6)	47 (52.2)	–
Rapid tumor growth	164 (19.9)	163 (55.4)	120 (18.2)	65 (72.2)	–
Suspicious sonography	77 (9.3)	237 (80.6)	30 (4.6)	77 (85.6)	–

Data represented as mean (SD) or median (lower – upper quartile) or n (%); LDH: serum lactate dehydrogenase, NLR: neutrophil-to-lymphocyte ratio, PLR: platelet-to-lymphocyte ratio

^a^ Applies to premenopausal women only

^b^ Applies to postmenopausal women only

^c^ Low abdominal pain, feeling of abdominal pressure or pollakiuria

## Results

Given the sample size of training data from 2010 to 2018 and testing data from 2019 to 2020, our validation study is based on a total number of 384 uLMS and 1485 LM. Table 1 shows the patient characteristics of the training and validation data stratified by tumor type. While uLMS cases were taken from the same counseling database with homogeneous characteristics between training and validation samples, we see higher LDH (median training vs. validation: 193 vs. 211 U/L), lower rates of symptoms of low abdominal pain, feeling of abdominal pressure or pollakiuria (49.5% vs. 4.2%), fewer suspicious sonographies (9.3% vs. 4.6%), but a higher rate of rapid tumor growth (40.6% vs. 49.6%) between LM samples.

**Fig. 2:**
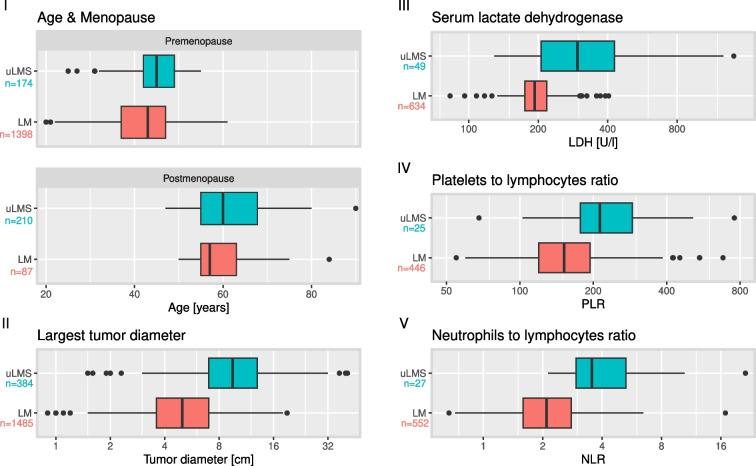
Continuous predictors of uterine leiomyosarcoma (uLMS) vs. leiomyoma (LM). Distributions are plotted with case numbers for: I Age; II Largest tumor diameter; III Serum lactate dehydrogenase; IV Platelet-to-lymphocyte ratio; V Neutrophil-to-lymphocyte ratio

When stratifying age by menopausal status, we do not notice any significant association of age with the tumor type in postmenopause (median age: LM 57 vs. LMS 60 years, $p=0.07$) as demonstrated in subplot I of Fig. 2. In premenopause, a significant age difference was found (median age: LM 43 vs. LMS 45 years, $p<0.001$). Substantial differences between uLMS and LM can be seen in tumor diameter, LDH, PLR and NLR with effect sizes ranging from –1.35 to –0.88 on the log-scaled values (see Fig. 2 II–V). Median tumor size is 5 cm in LM and 9.5 cm in LMS (Hedges’ $g=-1.32$, $p<0.001$), median serum LDH is 193 vs. 296 U/L (Hedges’ $g=-1.10$, $p<0.001$), median PLR is 152 vs. 213 (Hedges’ $g=-0.88$, $p<0.001$), median NLR is 2.09 vs. 3.55 (Hedges’ $g=-1.35$, $p<0.001$). Optimal cut-offs for distinguishing uLMS from LM are shown in Table 2. LDH represents the most specific blood marker with an average specificity of 94% (SD=0.04) and an odds ratio of 18.03 calculated for the threshold of 8.0 (SD=0.1) on log2-scale. This can be translated to a cut-off at 264 U/L. A good AUC can be achieved with tumor diameter (cut-off at 7.3 cm, average AUC=0.833, SD=0.016) and NLR (cut-off at 2.77, average AUC=0.853, SD=0.043). Cut-offs on the original scale are 187 for PLR and 298$\times 10^9/L$ for platelets.

**Table 2:** Optimal cut-offs and classification performance for continuous predictors to diagnose uLMS preoperatively

			Optimal	Bootstrap out-of-bag performances			
	Sample size		cutpoint	AUC	Sensitivity	Specificity	DOR
	LM	uLMS	[mean (SD)]	[mean (SD)]	[mean (SD)]	[mean (SD)]	
Age at surgery [years] in Premenopause	1398	174	42.6 (1.0)	0.610 (0.028)	0.709 (0.085)	0.478 (0.053)	2.22
Age at surgery [years] in Postmenopause	87	210	64.1 (2.8)	0.565 (0.046)	0.341 (0.102)	0.768 (0.140)	1.71
Tumor diameter [cm]	1485	384	7.3 (0.2)	0.833 (0.016)	0.732 (0.036)	0.803 (0.027)	11.15
log2(LDH) [*U*/*L*]	634	49	8.0 (0.1)	0.767 (0.061)	0.529 (0.105)	0.941 (0.040)	18.03
log2(NLR)	552	27	1.47 (0.13)	0.853 (0.043)	0.748 (0.160)	0.744 (0.067)	8.64
log2(PLR)	446	25	7.5 (0.1)	0.764 (0.073)	0.656 (0.171)	0.716 (0.065)	4.81
log2(Platelets) [$10^9/L$]	858	75	8.2 (0.1)	0.660 (0.045)	0.557 (0.151)	0.645 (0.102)	2.29

Take results with caution: blood markers were not collected systematically (selection bias can’t be ruled out)

LDH: serum lactate dehydrogenase, NLR: neutrophil-to-lymphocyte ratio, PLR: platelet-to-lymphocyte ratio, DOR: Diagnostic odds ratio

**Fig. 3:**
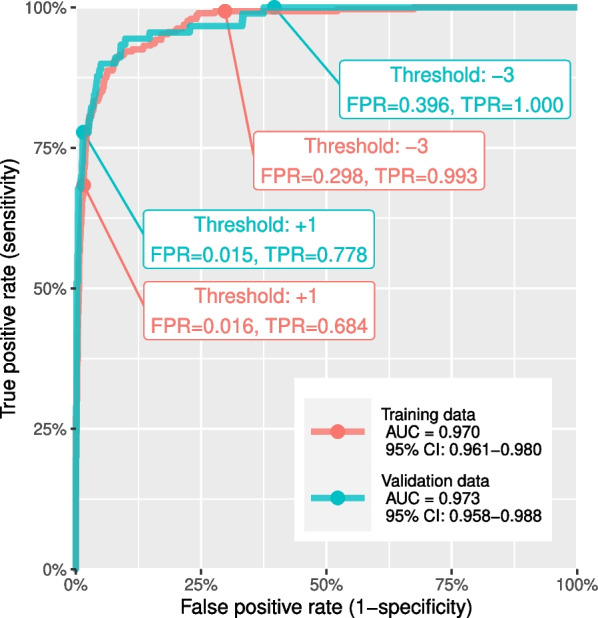
Receiver operating characteristic of pLMS applied to training and validation data. False positive (FPR) and true positive rates (TPR) vary at the proposed diagnostic thresholds at −3 and +1, although the AUC values do not differ

**Fig. 4:**
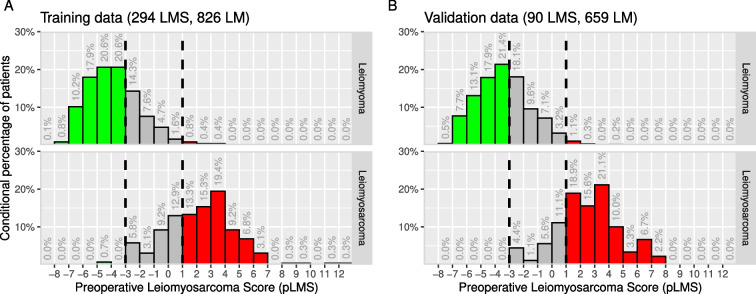
Distribution of pLMS in the training and validation datasets. Tumor classification in the validation set is still good, but there is a slight shift in both, LM and LMS, towards higher scores can be seen. Threshold calibration may be required

The application of the pLMS score system to the unseen validation data resulted in comparable AUC values (training AUC=0.970 vs. validation AUC=0.973, see Fig. 3). When analyzing the specificity and sensitivity at the proposed cut-offs at −3 and +1, a higher false positive rate (FPR) was seen at −3 cut-off (validation FPR=39.6% vs. training FPR=29.8%) with equivalent true positive rate (TPR) and a higher TPR was seen at the +1 cut-off (validation TPR=77.8% vs. training FPR=68.4%) with equivalent FPR. The effect is due to a shift in the score distributions of both, LM and LMS cases, as shown in Fig. 4.

**Fig. 5:**
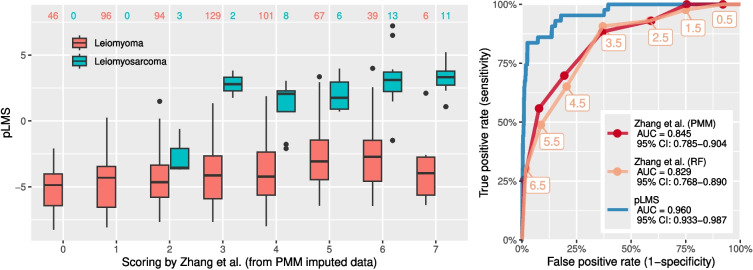
Comparison and correlation of the scoring systems by Zhang et al. and Köhler et al. (pLMS). Left: Absolute number of LM and uLMS at each Zhang score based on imputed data using predictive mean matching (PMM) with boxplots of pLMS. Right: ROC curves with DeLong confidence intervals for the AUC for pLMS and Zhang score based on PMM imputed data and random forest imputed data (RF). The labels indicate the diagnostic cut-off for the Zhang score

pLMS was compared with the predictive performance of the Zhang et al. score system in a dataset consisting of 43 uLMS and 578 LM after imputation of a maximum of one missing value of age, tumor diameter, NLR, LDH or platelets. Within uLMS 16 missing LNR, 8 missing LDH and 2 missing platelet values were imputed. Within LM 144 NLR, 3 LDH and 108 platelet values were imputed. The patient score by Zhang et al. ranging from 0 to 7 was obtained by summing the subscores (1 point if age $\ge 40$ years, 2 points if tumor diameter $\ge$7 cm, 1 point if NLR $\ge 2.8$, 2 points if LDH $\ge 193$ U/L, 1 point if platelets $\ge 298 \times 10^9/L$). The score system of Zhang et al. achieved a sensitivity of 0.88 (38/43) and specificity of 0.63 (365/578) using the PMM imputed dataset at the proposed cut-off of 3.5 ($\ge 4$). As shown in Fig. 5, AUC was 0.845 (95%-CI: 0.785–0.904) which is according to DeLong confidence intervals significantly lower than pLMS with 0.960 (95%-CI: 0.933–0.987). Especially at scores ranging between 3 and 7, pLMS is able to better discriminate uLMS from LM. We also see that the majority of LM with a Zhang score ${\ge }4$ (74.1%) have pLMS values less than −1, which would reduce the number of LM falsely classified as uLMS (false positives).

**Fig. 6:**
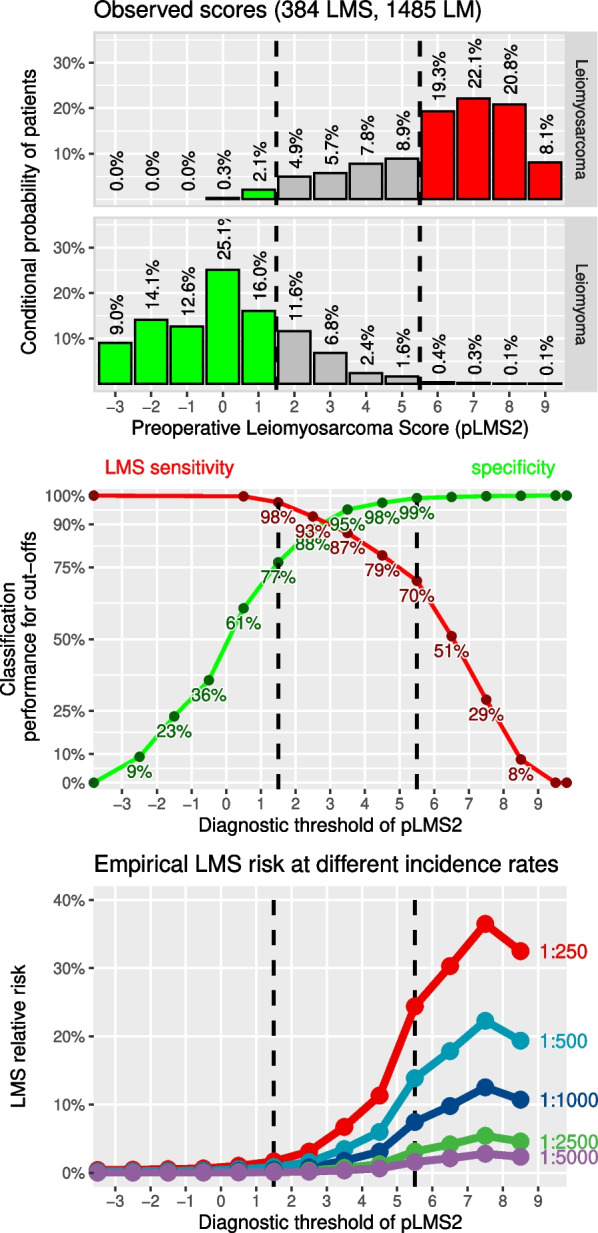
Score distribution of the integer score system pLMS2 and LMS relative risk. Empirical relative risk is demonstrated at different prevalence rates, leading to proposed diagnostic thresholds of 1.5 and 5.5

Logistic regression was performed on all samples (training and validation) to create an updated score based on integer subscores. For this purpose, age was categorized using the optimal cut-offs for premenopausal and postmenopausal cases and included only in interaction with menopausal status. Tumor diameter was categorized into three intervals at optimal cut-offs calculated for 7.5 and 11 cm. Solitary tumor, symptoms, and failed medical or interventional therapy of myoma dropped from the model equation. The beta coefficients derived from the final model were then used to determine a factor *c* to obtain reasonable integers without much loss of model accuracy. Because the coefficients were close to integers, the coefficient *c* was close to 1 with the integer subscores obtained from the regression as shown in Table 3. The new pLMS2 score system for preoperative uLMS assessment has subscores between −2 and +3. pLMS2 is the sum of all sub-scores that apply to a preoperative patient. The minimum pLMS2 is −3 (in premenopause with hypermenorrhea and dysmenorrhea) and the maximum value is +9 (in postmenopause with bleeding, rapid tumor growth with a diameter $\ge$11 cm and suspicious sonography). Sensitivity and specificity were obtained by applying the new clinical score to the LM and uLMS data. The classification performance is shown in Fig. 6. The empirical relative risk is demonstrated at different prevalence rates, leading to the proposed diagnostic thresholds for pLMS2 of 1.5 and 5.5. The first threshold of 1.5 is associated with a high sensitivity of 98% and a moderate specificity of 77%, while the second threshold of 5.5 is associated with a high specificity of 99% and a moderate sensitivity of 70%.

**Table 3:** Final model of the multivariable logistic regression with integer scores for pLMS2

		$\beta$	SD	P	Subscores for pLMS2	OR	(95%-CI of OR)
Intercept		−4.14	0.26		0		
Premenopause	Age<43 years	–			0		
Premenopause	Age$\ge$43 years	0.57	0.24	0.017	1	1.78	(1.11–2.85)
Postmenopause	any Age	2.15	0.29	<0.001	2	8.56	(4.86–15.06)
Tumor diameter	<7.5 cm	–			0		
	$\ge$7.5 cm & <11 cm	0.99	0.22	<0.001	1	2.70	(1.75–4.17)
	$\ge$11 cm	2.10	0.28	<0.001	2	8.21	(4.75–14.17)
Intermenstrual bleeding^a^		1.84	0.26	<0.001	2	6.27	(3.76–10.46)
Hypermenorrhea^a^		−0.96	0.25	<0.001	−1	0.38	(0.23–0.63)
Dysmenorrhea^a^		−2.10	0.37	<0.001	−2	0.12	(0.06–0.26)
Postmenopausal bleeding^b^		1.03	0.33	0.002	1	2.81	(1.48–5.37)
Rapid tumor growth		0.97	0.21	<0.001	1	2.64	(1.76–3.94)
Suspicious sonography		2.95	0.20	<0.001	3	19.11	(12.88–28.35)

^a^ Applies to premenopausal women only

^b^ Applies to postmenopausal women only

An online tool for the calculation of pLMS2 is available for research purposes at http://kaderali.org/LMS.

## Discussion

Morcellation of incidentally discovered uLMS remains a challenging problem in the management of this entity. Although current evidence suggests that morcellation does not affect overall survival, it must be accepted that it shortens the locoregional recurrence-free interval and increases the number of locoregional recurrences [8, 9]. The latter serious drawback can only be largely offset by adequate preoperative diagnosis and improved differentiation between uterine LM and uLMS. To this end, we published in 2019 a diagnostic risk score called pLMS [14] with satisfactory discriminatory power to distinguish uLMS from LM. The study was based on a large multicenter cohort of 826 LM and 293 uLMS. In the present study, we have validated this score in 90 newly included and predominantly retrospectively collected uLMS and 659 prospectively collected LM. Between the primary and validation groups, the FPR and TPR of pLMS differed at the proposed diagnostic thresholds of −3 and +1, although the AUC values did not differ, which constitutes an important finding to assess the generalizability and the applicability on new unseen patient data. For this reason, we decided to update pLMS based on both datasets, which include a total of 384 uLMS and 1485 LM. To our knowledge, this is still the largest case-control study worldwide for this rare uterine sarcoma. However, the still predominantly retrospective collection of uLMS data remains a limitation of this analysis. Due to the extreme rarity of uLMS, a prospective analysis is unlikely in the next 10 years. The main advantage of this low-cost risk score is that all variables can be easily collected by anamnesis, clinical findings together with sonography, eliminating the need for complex and expensive imaging procedures (computed tomography [CT], MRI, positron emission tomography–computed tomography [PET/CT]) at the screening stage. In our first publication, we provided a comprehensive account of the sonographic criteria for uLMS [14]. Additionally, ultrasonographic features have been documented in a review article [35]. The problem of so-called rapid growth was also discussed there. However, in pLMS2, rapid growth only has a subscore value of 1, so a misjudgment does not have an overly significant impact on the overall score (see Table 3).

In the proposed score, the diagnostic thresholds for pLMS2 are 1.5 and 5.5. The first threshold of 1.5 has a sensitivity of 98% and a specificity of 77%. It is therefore expected that 77% of LM will have a pLMS2 $\le 1$, while only 2% of uLMS will have a pLMS2 $\le 1$. The second threshold of 5.5 with a specificity of 99% and a sensitivity of 70% means that only 1% of LMs can be expected to have a pLMS2 $\ge 6$, while this is the case for 70% of uLMS’. The risk of uLMS and LM to the left and right of these thresholds can therefore be estimated with sufficient confidence. For patients with intermediate scores between 1.5 and 5.5, we recommend further cost-efficient diagnostics. Based on our available data, we examined appropriate cutoff values for blood markers such as LDH, NLR and PLR, which were 264 U/L, 2.77 and 187$\times 10^9/L$, respectively. LDH measurement is also an component of rPRESS [24] that was highly specific at a cutoff value of 279 U/L. Interestingly, the median LDH concentration in degenerated LM reported by Suh et al. [19] was 196.5 U/L, which is very close to the threshold proposed by Zhang et al. (193 U/L) and can be directly translated into a weak specificity of about 50%. Therefore, the LDH threshold of 339.5 U/L was set much higher by Suh et al. The AUC of their reported combination of NLR, LDH and age was 0.86.

Furthermore, we noticed that the AUC of Zhang’s scoring system exhibited a slight reduction when missing data were imputed with RF as opposed to PMM. Given the recent literature’s preference for PMM [36], it can be postulated that the imputed values may be more accurately represented on an unbiased scale, therby yielding better results. Although the performance of Zhang’s score at the proposed cut-off achieved a sensitivity of 88% and a specificity of 63%, which is in close proximity to the performance published in the original paper (80% sensitivity and 78% specificity), the chosen imputation methods limit the interpretation of the diagnostic performance. To achieve further validation, prospective collection of paraclinical variables is needed.

However, it is important to note that higher blood markers correlate with larger tumor diameter and thus, in principle, with a higher score. In the lower score range, the laboratory values are also of limited significance. In women with a low interval score and regular blood values, or if further diagnostic clarification is required, further imaging with pelvic MRI should be performed if conservative surgery is urgently required, which is in most cases inadequate. MRI was not part of our study. However, on MRI, tumors are considered suspicious for the presence of uLMS with the following signal intensity (SI) characteristics on native $\text {T}_1$-weighted imaging (T1W): intermediate or low SI (especially cystic necrosis), scattered areas of high SI (especially hemorrhage). In T2W: SI mainly intermediate, with heterogeneous hypo- (hemorrhage) and hyperintense SI, some areas of very high SI (necrosis, cysts). In T1W contrast-enhanced: heterogeneous enhancement, weaker enhancement than homogeneously enhancing myometrium, necrosis and cysts show no enhancement, often with irregular margins [37–39]. In retrospective cohort studies, a high sensitivity (95–100%) and specificity (97%–100%) for the detection of LMS was found [40, 41]. Overall, pelvic MRI, especially in combination with suspicious clinical, hematologic findings, or radiomic features may further increase diagnostic certainty [42]. PET/CT is also suggested to further narrow down the diagnosis [43]. Transcervical core needle biopsy can only be applied in order to achieve clarification in cases in which the diagnosis of uterine masses remains uncertain despite having performed all of the above-mentioned diagnostic measures [44, 45]. However, this is definitely not a diagnostic method for intermenstrual and postmenopausal bleeding. Nevertheless, the experience with this approach in selected cases was surprisingly positive: of 372 biopsies, 18 had a needle biopsy score $\ge 1$, of which 7 were confirmed sarcomas, as shown by Kawamura et al. [45]. In contrast to transcervical core needle biopsy, endometrial biopsy is indicated only in cases of postmenopausal or abnormal uterine bleeding or in the presence of pathological intracavitary findings on sonography. Even in these cases, the specific preoperative histologic diagnosis by endometrial biopsy was correct in 30.0% of LMS [46].

Another sophisticated method for diagnosing uLMS is through the analysis of genomic landmark alterations derived from targeted next-generation sequencing of peripheral blood samples [47]. This approach has been shown to be effective in identifying uLMS cases, with 80% of cases exhibiting more than one genomic alteration in specific genes, including TP53, RB1, ATRX, PTEN, CDKN2A, and MDM2.

## Conclusions

The validation of pLMS shows a stable discriminatory ability as expressed by AUC, but caution is required with the cut-off values, as sensitivity and specificity vary. On new validation data from German residents, the rating system of Zhang et al. performs weaker than pLMS using either imputation method. The updated clinical score pLMS2 is based solely on anamnesis, clinical findings and vaginal ultrasound performed at every gynecologic examination of a LM (see Table 3). Data collection is simple and convenient, with no additional cost. The proposed reasonable thresholds of 1.5 and 5.5 provide guidance to prevent unnecessary or inappropriate surgery and to make the use of further diagnostic measures cost-efficient. For cases with an intermediate score between 1.5 and 5.5, the determination of LDH, NLR, and PLR can be used to provide further evidence. Pelvic MRI and, only in a few exceptional cases, a transcervical punch biopsy are indicated only when diagnostic uncertainty remains.

## Data Availability

Individual patient information cannot be shared. Anonymization cannot fully guarantee that individual patients are indirectly non-identifiable. Aggregated results, as they are already given in tables and figures, and more detailed statistical characteristics can be transmitted on reasonable request.

## Abbreviations

AUC: Area under the receiver operating characteristic curve
CA125: Cancer antigen 125
CT: Computed tomography
DKSM: German Clinical Center of Excellence for Genital Sarcomas and Mixed Tumors
DOR: Diagnostic odds ratio
FPR: False positive rate
HE4: Human epididymis protein 4
LDH: Serum lactate dehydrogenase
LM: Leiomyoma
MRI: Magnetic resonance imaging
NLR: Neutrophil-to-lymphocyte ratio
PET/CT: Positron emission tomography–computed tomography
PLR: Platelet-to-lymphocyte ratio
PMM: Predictive mean matching
ROC: Receiver operating characteristic
RF: Random forest
rPRESS: Revised preoperative sarcoma score
SD: Standard deviation
SI: Signal intensity
T1W: T_1_-weighted imaging
T2W: T_2_-weighted imaging
TPR: True positive rate
uLMS: Uterine leiomyosarcoma
WBC: White blood cell count

## Footnotes

1 $\lfloor .\rceil$ denotes rounding to the nearest integer.
